# A halophilic metalloprotease from *Salinivibrio* sp. YH4 and its application in antioxidant peptide production

**DOI:** 10.3389/fmicb.2025.1595109

**Published:** 2025-05-19

**Authors:** Dan Liu, Yuyang Xiao, Yingying Wei, Maojia Xie, Yu Huang, Chaoyu Gan, Hailun He

**Affiliations:** ^1^Guangxi Colleges and Universities Key Laboratory of Biological Molecular Medicine Research, Department of Biochemistry and Molecular Biology, School of Pre-Clinical Medicine, Guangxi Medical University, Nanning, Guangxi, China; ^2^School of Life Sciences, Central South University, Changsha, Hunan, China; ^3^Xiangya School of Medicine, Central South University, Changsha, Hunan, China

**Keywords:** *Salinivibrio*, metalloprotease, collagen hydrolysis, antioxidant, oxidative stress

## Abstract

**Purposes:**

This study aimed to develop a sustainable strategy for valorizing protein-rich industrial by-products into functional antioxidants using halophilic biocatalysts, addressing environmental challenges and the demand for bioactive compounds.

**Methods:**

A moderately halophilic bacterium, *Salinivibrio* sp. YH4, was isolated from Yuncheng Salt Lake and identified as *S. costicola* (99% 16S rRNA homology). The extracellular protease EYHIII? was purified and biochemically characterized for thermal/pH stability, halotolerance, and substrate specificity. Fish collagen hydrolysates generated by EYHIII were evaluated for antioxidant capacity via 1,1-diphenyl-2-picrylhydrazyl (DPPH), hydroxyl, and peroxyl radical scavenging assays. Cellular bioactivity was validated in high glucose-stressed human umbilical vein endothelial cells (HUVECs), analyzing ROS levels and antioxidant enzyme activity.

**Results:**

EYHIII was a thermostable (5060°C) and alkaliphilic (pH 7.59.5) M4-family metalloprotease. The enzyme retained >80% activity under high salinity conditions (1 M NaCl) and exhibited strict substrate specificity for hydrophobic residues (Phe/Leu) at the P1’ position. It efficiently hydrolyzed both soluble and insoluble collagens. Fish collagen hydrolysates generated by EYHIII demonstrated potent antioxidant activity, scavenging 33.53 ± 3.30% of DPPH radicals and 45.55 ± 3.00% of hydroxyl radicals at 3 mg/mL, with a peroxyl radical absorbance capacity of 1.69 ± 0.07 mmol TE/g. In human umbilical vein endothelial cells (HUVECs), the hydrolysate reduced high glucose-induced reactive oxygen species (ROS) to baseline levels at 200 μg/mL. It also significantly upregulated antioxidant enzymes compared to damaged controls: superoxide dismutase (SOD, 103.55%), catalase (CAT, 110.96%), and glutathione peroxidase (GSH-Px, 135.79%) (all *P* < 0.05).

**Conclusions:**

This study highlighted Salinivibrio sp. YH4 and its protease EYHIII as a sustainable platform for converting collagen waste into high-value antioxidants. These findings addressed both environmental pollution and the growing demand for functional bioactive compounds. The results underscored the potential of halophilic biocatalysts in advancing circular economy strategies for protein resource utilization.

## 1 Introduction

The sustainable utilization of protein-rich industrial by-products, particularly those abundant in collagen, has emerged as a critical challenge in biotechnology (Qiu et al., 2022; Chen and Wang, 2024; Cheng et al., 2024). Collagen-rich materials from meat, poultry, seafood, and fish processing are often underutilized or discarded, leading to environmental degradation and wasted valuable protein resources (Salim et al., 2024; Srinivasan et al., 2025). Transforming these materials into value-added products is essential for both environmental protection and efficient resource use.

Enzymatic hydrolysis offers an eco-friendly solution by converting collagen into bioactive peptides with antioxidant, antimicrobial, antihypertensive and anti-aging properties (Chang et al., 2024, Gao et al., 2024, Santos Filipe et al., 2024, Zhang et al., 2025). However, current industrial processes rely heavily on commercial proteases, such as trypsin and papain, which perform poorly under extreme conditions like high pH, high salt concentrations, and elevated temperatures. These limitations restrict their applicability in food processing and environmental remediation (Jin et al., 2019; Sujitha and Shanthi, 2023).

Metalloproteases have attracted significant research interest owing to their remarkable stability under extreme conditions and versatile catalytic capabilities (Rehman et al., 2017; Mushtaq et al., 2024; Li et al., 2025). These enzymes can break down complex protein substances, even in difficult conditions, which makes them very useful for industry. Examples of commercial metalloproteases include Thermoase PC10F and Protin PC10F (Amano Enzyme Inc., Japan), Neutrase (Novo Nordisk, Denmark), and the thermostable Boilysin variant (developed in Groningen, The Netherlands). These enzymes are widely used in industries such as food processing, pharmaceuticals, brewing, leather production, photographic film processing, and baking (Wu and Chen, 2011). Despite their extensive applications, the potential of metalloproteases in collagen waste valorization remains underexplored.

Microbial extracellular enzymes, particularly those derived from extremophiles, show unique industrial potential. These microorganisms thrive in harsh environments, which enabled their enzymes to maintain stability under these conditions, made them promising biocatalysts (Ruginescu et al., 2022; Berezina et al., 2024). Moderately halophilic bacteria, such as those in the genus *Salinivibrio*, exemplifies this potential (Tao et al., 2021; Yavari-Bafghi and Amoozegar, 2025). These bacteria thrive in high-salinity environments, exhibit low pathogenicity, and secrete diverse hydrolytic enzymes (e.g., proteases, chitinases, lipases), positioning them as an underexplored resource for biocatalysis and bioremediation (John et al., 2019; Tao et al., 2021; Ruginescu et al., 2022). Recent advances highlight *Salinivibrio*’s enzymatic versatility. For instance, chitinases from *Salinivibrio* sp. BAO-1801 degrade chitin waste into chitobiose, enabling the circular valorization of marine by-products (Le and Yang, 2018). Similarly, serine proteinases and metalloproteinases from *Salinivibrio* strains exhibit robust activity in organic solvents and imidazolium-based ionic liquids, offering novel avenues for industrial biocatalysis (Badoei-Dalfard et al., 2018). However, while metalloproteases from *Salinivibrio* have been noted for their activity in organic solvents and ionic liquids, their role in collagen waste valorization has not been thoroughly investigated.

Here, we reported the discovery of *Salinivibrio* sp. YH4, a protease-producing strain isolated from the hypersaline sediments of Yuncheng Salt Lake. We characterized its dominant extracellular protease, EYHIII, a halotolerant metalloprotease of the M4 family, and demonstrated its catalytic properties on various substrates, as well as its ability to hydrolyze low-value collagen into antioxidant peptides. Unlike conventional proteases, EYHIII retained >80% activity at 1 M NaCl and >90% activity across a pH range of 7.0 to 10.0, aligning with the demands of alkaline and saline industrial environments. Our findings indicated that *Salinivibrio* sp. YH4 can serve as a halophilic chassis for sustainable collagen waste valorization by producing antioxidant peptides with dual bioactivity. By aligning enzymatic properties with industrial demands, this work advanced the circular bioeconomy paradigm, transforming environmental liabilities into health-promoting resources.

## 2 Materials and methods

### 2.1 Bacterial strain and identification

Strain YH4 was isolated from a mud sample collected from Yuncheng Salt Lake, Shanxi Province, China. Culturable bacterial strains were initially enriched using modified LB medium (10% NaCl, 1% tryptone, 0.5% yeast extract, pH 7.2) under aerated conditions at 30°C with 200 rpm for 24 h. Protease-production strains were subsequently screened on selective skim milk agar plates (10% NaCl, 0.5% peptone, 0.1% yeast extract, 1% skim milk, 1.5% agar) incubated at 37°C for 48 h. Strain YH4 was identified based on its proteolytic activity, characterized by distinct hydrolysis zones on the skim milk agar medium. Genomic DNA was extracted using a bacterial genomic DNA extraction kit, and the 16S rRNA gene was amplified via polymerase chain reaction (PCR) with universal primers 27F and 1492R (Wang et al., 2020). The amplified product was sequenced by BGI Co., Ltd. (China) for taxonomic identification.

To evaluate halotolerance, strain YH4 was inoculated into fresh LB medium containing NaCl concentrations ranging from 0 to 5 M at a 2% (v/v) inoculum density. Cultivation was conducted in 30°C with 200 rpm. Growth was monitored after 24 h of incubation via optical density measurements at 600 nm (OD₆₀₀) to assess salt-dependent viability.

### 2.2 Extracellular protease profile analysis of YH4

The activated strain was inoculated into a fermentation medium for enzyme production, containing 2% corn flour, 1% wheat bran, 2% soybean meal, 0.1% Na₂HPO₄, 0.03% KH₂PO₄, 0.1% CaCl₂, 0.1% Na₂CO₃, and 100 mL artificial seawater (pH 7.2). The culture was prepared with a liquid volume of 25 mL per 250 mL flask and incubated at 30°C with 200 rpm for 1–5 days. Crude enzyme solutions were harvested at daily intervals to assess protease production over time.

Extracellular protease profile was detected using casein as the substrate via the substrate immersion enzyme assay (Liu et al., 2015). To classify protease types, inhibitors phenylmethylsulfonyl fluoride (PMSF, 2.5 mM) and 1,10-phenanthroline (OP, 2.5 mM) were incorporated into the substrate solution, and residual enzyme activity was measured according to the method described by Liu et al. (2019b).

### 2.3 Purification and identification of protease EYHIII

Following fermentation at 30°C with 200 rpm for 96 h, the broth was centrifuged at 12,000 × *g* for 30 min to isolate the supernatant as the crude enzyme solution. Ammonium sulfate ((NH_4_)_2_SO₄) was added to the supernatant to achieve 50% (w/v) concentration, followed by overnight precipitation at 4°C. The precipitated protein was collected via centrifugation (12,000 × *g*, 30 min), redissolved in 20 mM Tris–HCl buffer (pH 8.0), and dialyzed (MWCO 10 kDa) to remove low-molecular-weight impurities. The dialyzed sample was loaded onto a HiTrap Capto DEAE column (5 mL; GE Healthcare, United States) pre-equilibrated with 20 mM Tris–HCl buffer (pH 8.0) using an NGC chromatography system (Bio-Rad, USA). Bound proteins were eluted with a linear NaCl gradient (0–1.0 M) in the same buffer at a flow rate of 1 mL/min. Fractions exhibiting protease activity were pooled and further purified by gel filtration chromatography on a Superdex 75 Increase 10/300 GL column (24 mL; GE Healthcare, United States) at a flow rate of 0.5 mL/min. High-activity fractions were collected, and the molecular weight and purity of the protease were verified via SDS-PAGE under reducing conditions. The purified enzyme, designated EYHIII, was quantified using a BCA protein assay kit (Beyotime, China) with bovine serum albumin (BSA) as the standard.

For structural identification, the excised SDS-PAGE band corresponding to EYHIII was submitted to Sangon Biotech (Shanghai, China) for LC–MS/MS analysis. The acquired mass spectrometry data were aligned against the NCBI non-redundant protein database to identify homologous sequences. Conserved domains were identified, and gene-specific primers (Table 1) were designed to amplify flanking regions via thermal asymmetric interlaced PCR (TAIL-PCR). The full-length EYHIII gene sequence was assembled from overlapping PCR products and validated by sequencing. The tertiary structure of EYHIII was predicted using the homology modeling tool Phyre2.^1^

**Table 1:** The sequence of primers.

Name		Sequence (5′–3′)
EYHIII Tail-PCR	Adn	GCAGCGTTA
	Sp1n	GACCTTCCAGTTGACGATGCGTGT
	Sp2n	CCACGAAGTCAGTCACGGTTTCAC
	Sp3n	TGGCGACGTAGACTGGATTGTCGG
	Sp1c	CCGACAATCCAGTCTACGTCGCCA
	Sp2c	GTGAAACCGTGACTGACTTCGTGG
	Sp3c	ACACGCATCGTCAACTGGAAGGTC
	Adc	AAKYRTATG
EYHIII	F	ATGAAATTATCCAAGTTGACTTG
	R	GTTGCGAACCAAGCTTACACC

### 2.4 Effects of temperature, pH, NaCl, and metal ions on the activity and stability of EYHIII

To determine the optimal temperature for EYHIII activity, the enzyme was diluted appropriately and mixed with 2% (w/v) casein in a 1:1 (v/v) ratio. The mixtures were incubated at temperatures ranging from 30 to 80°C for 10 min, and enzyme activity was quantified using the Folin phenol method (Liu et al., 2019a,b). All optimal condition tests (thermal/pH/salt) utilized the condition exhibiting maximum activity as the baseline control (100% activity), thereby enabling the calculation of relative activities at other conditions. For thermal stability assessment, EYHIII solutions were incubated at 50, 60, and 70°C for intervals of 10–60 min. In the experiment of optimum enzyme activity temperature and pH value, the enzyme activity of protease was high at 50°C and pH8.0. And pre-tests showed <5% activity deviation at 50°C/pH 8.0 across 3 biological replicates. Thus, all stability tests (thermal/pH/salt) used the same baseline control (untreated enzyme at 50°C/pH 8.0) to ensure cross-experiment comparability.

The pH optimum of EYHIII was evaluated by assaying activity at 50°C across a pH gradient (4.0–12.0) using the following 0.02 M buffers: Citrate-Na₂HPO₄ (pH 4.0–7.0), Tris–HCl (pH 7.0–9.0), and Gly-NaOH (pH 9.0–12.0). For pH stability analysis, the enzyme was pre-incubated in these buffers at 4°C for 24 h, and residual activity was measured under standard conditions (50°C, pH 8.0).

Halotolerance was assessed by incubating EYHIII in NaCl solutions (0–4 M) at 4°C for 24 h, followed by residual activity measurement at 50°C and pH 8.0. The influence of NaCl on instantaneous activity was similarly tested without pre-incubation.

To evaluate metal ion effects, EYHIII was incubated for 10 min with 2.5 mM or 10 mM solutions of K^+^, Ca^2+^, Na^+^, Ba^2+^, Co^2+^, Mn^2+^, Mg^2+^, Al^3+^, Cu^2+^, Zn^2+^, Fe^3+^, or Fe^2+^. Activity in the absence of metal ions was defined as 100%. All assays were conducted at 50°C and pH 8.0.

### 2.5 Substrate specificity of protease EYHIII

The substrate specificity of EYHIII was determined by measuring its hydrolytic activity toward the following substrates: gelatin, casein, azocasein, synthetic dipeptides, soluble collagen, and insoluble collagen. Gelatin, casein, and elastin were purchased from Sigma-Aldrich (United States). Soluble collagens were extracted from porcine and salmon skins as described by Wu et al. (2018). Insoluble collagen substrates—bovine collagen fiber BF-50, porcine collagen PP-100, and bovine collagen BP-SF—were provided by Shanghai Sidao Yilang Investment Management Co., Ltd. (China). Synthetic dipeptide substrates (FA-Gly-Leu-NH₂, FA-Gly-Phe-NH₂, FA-Gly-Val-NH₂, FA-Lys-Ala-OH, FA-Ala-Arg-OH, FA-Glu-Glu-OH) were synthesized by Suzhou QiangYao Biotechnology Co., Ltd. (China).

Casein hydrolysis: Proteolytic activity was quantified using the Folin–Ciocalteu method (Liu et al., 2019a).

Insoluble collagen and gelatin hydrolysis: Activities were measured according to Wu et al. (2017). One unit (U) of enzyme activity was defined as the amount of enzyme releasing 1 μmol leucine from collagen per hour or 1 μmol tyrosine equivalents from gelatin per minute.

Azocasein hydrolysis: A reaction mixture containing 40 μL of EYHIII and 40 μL of 2% (w/v) azocasein was incubated at 50°C for 10 min. The reaction was terminated with 80 μL of 10% (w/v) trichloroacetic acid, centrifuged at 12,000 × *g* for 1 min, and 100 μL of the supernatant was mixed with 100 μL of 0.5 M NaOH. Absorbance was measured at 400 nm, with one unit defined as the enzyme causing a Δ*A*_400_ of 0.01 per minute (Coelho et al., 2016).

For soluble collagen substrates (salmon and porcine), 2–4 μL of EYHIII (0.2 mg/mL) was incubated with 20 μL of collagen (5 mg/mL) at 50°C for 10–30 min. Reactions were terminated by adding 5 μL denaturing buffer, boiling for 5 min, and analyzing degradation patterns via SDS-PAGE (12% gel, Coomassie staining) (Wu et al., 2018).

The synthetic dipeptide substrate was diluted to 1 mM in 20 mM Tris–HCl buffer (pH 8.0). EYHIII was similarly diluted in the same buffer to ensure consistent experimental conditions. A 100 μL of EYHIII was mixed with 100 μL of the dipeptide substrate, and the mixture was transferred to a cuvette in an Agilent Cary 60 UV–Vis spectrophotometer. After a 30 s equilibration period, absorbance changes were monitored in real time using the instrument’s Time Course Measurement software. The 200 μL reaction system maintained a final substrate concentration of 0.5 mM (1% DMSO) and was analyzed at 25°C for 600 s, with data points recorded at 1 s intervals (Xie et al., 2009).

The catalytic efficiency (*k_cat_*/ *K_m_*) was derived from the linear decrease in absorbance at 345 nm, which reflects the reaction velocity. Under the assumption [*S*]_0_<<*K_m_*, the following relationship was applied:


$$k_{\mathrm{cat}}/K_{m}=v/({\left[E\right]}_{0}.{\left[S\right]}_{0})=k/(b.\Delta \varepsilon 345)/({\left[E\right]}_{0}.{\left[S\right]}_{0})$$

where:

*K_cat_*/*K_m_*:Apparent second-order rate constant, s^−1^·M^−1^.

*v*: Enzymatic reaction rate, M·s^−1^.

*k*: Absolute slope of the linear absorbance decline at 345 nm, s^−1^.

*b*: Cuvette path length, 1 cm.

∆*ε*345: Molar extinction coefficient, −317 M^−1^ cm^−1^.

[*E*]_0_: Initial enzyme concentration, M.

[*S*]_0_: Initial substrate concentration, M.

### 2.6 Antioxidant activity assay of collagen hydrolytic peptides

The antioxidant activity of collagen hydrolytic peptides was assessed using three established assays: DPPH (1,1-diphenyl-2-picrylhydrazyl) radical scavenging activity, hydroxyl radical scavenging activity, and oxygen radical absorbance capacity (ORAC). These assays were performed according to methodologies described by Liu et al. (2019a) and Xiao et al. (2024), with minor adaptations to optimize conditions for collagen-derived peptides.

#### 2.6.1 DPPH radical scavenging assay

A clean 0.5 mL centrifuge tube was prepared. For the experimental group, 40 μL of collagen hydrolytic peptides was added, while 40 μL of double-distilled water (ddH_2_O) was used for the control group. Subsequently, 200 μL of 0.1 mM DPPH working solution was added to each tube. The tubes were incubated at 37°C for 60 min under dark conditions. After incubation, 150 μL of the reaction mixture was transferred to a microplate, and absorbance at 517 nm was measured using an Enspire spectrophotometer (Perkin Elmer, Waltham, MA, USA). The assay was repeated three times for accuracy. The DPPH scavenging rate (%) was calculated using the following formula:


$$\text{Scavenging rate}(\%)=[1-\frac{A_{\text{sample}}}{A_{\text{control}}}]\times 100\%$$

#### 2.6.2 Hydroxyl radical scavenging assay

Forty microliiter of FeSO_4_ (2 mM), 40 μL of OP (2 mM), and 80 μL of t collagen hydrolytic peptides were mixed. The reaction was initiated by adding 40 μL of H_2_O_2_ solution (0.1% v/v). The tubes were incubated at 37°C for 60 min under dark conditions. Following incubation, 150 μL of the reaction mixture was transferred to a microplate, and absorbance at 536 nm was measured. Damage group controls (without antioxidant) and blank group controls (without H₂O₂) were included. The assay was repeated three times to ensure reliability. The hydroxyl radical scavenging rate (%) was calculated as:


$$\text{Scavenging rate}(\%)=\frac{A_{\text{sample}}-A_{\text{damage}}}{A_{\text{blank}}-A_{\text{damage}}}\times 100\%$$

#### 2.6.3 ORAC activity assay

In a 96-well plate, 150 μL of sodium fluorescein solution (96 nM in PBS, pH 7.4) was added to each well. For the blank control, 20 μL of 0.01 M PBS was introduced, while 20 μL of collagen hydrolytic peptides and 20 μL of Trolox solution (150 μg/mL) were added to the experimental and positive control groups, respectively. The reaction was initiated by adding 30 μL of preheated AAPH solution (320 mM at 37°C). Fluorescence was monitored at 30-s intervals for 150 min with excitation/emission wavelengths set to 485/538 nm.

### 2.7 Cytotoxicity of collagen hydrolytic peptides and intracellular reactive oxygen species determination

Human umbilical vein endothelial cells (HUVECs) were seeded in 96-well plates at a density of 1 × 10^5^ cells/mL in RPMI-1640 medium supplemented with 10% (v/v) fetal bovine serum (FBS) and incubated for 12 h at 37°C under 5% CO₂. Cells were treated with collagen hydrolysate peptides (100–600 μg/mL) for 12 h. After treatment, 10 μL of MTT solution (5 mg/mL) was added to each well, followed by a 4 h incubation. The medium was removed, and 150 μL of DMSO was added to each well. Plates were agitated on an orbital shaker (10 min, low speed), and then absorbance was measured at 490 nm to evaluate cell viability (Liu et al., 2019a).

HUVECs (1 × 10^5^ cells/mL) were seeded in 24-well plates and cultured for 12 h. Cells were washed thrice with PBS and incubated for 12 h in RPMI 1640 containing 35 mM glucose and hydrolysate peptides (50–300 μg/mL). DCFH-DA (2,7-dichlorofluorescein diacetate, 1:1000 dilution in RPMI 1640) was added, and cells were incubated for 1 h. Excess probe was removed by washing with 10 mM PBS. Fluorescence intensity (indicative of ROS levels) and cell morphology were analyzed using a Nikon ECLIPSE TE2000-U fluorescence microscope (excitation/emission: 485/535 nm) (Wu et al., 2018; Liu et al., 2019a).

### 2.8 Intracellular antioxidant enzyme activity

Treated HUVECs were harvested, washed twice with ice-cold PBS, and lysed via ultrasonic homogenization (200 W, 3 s pulse/10 s interval, 8 cycles, ice bath). Lysates were centrifuged (12,000 × g, 15 min, 4°C), and supernatants were collected. The activities of superoxide dismutase (SOD), catalase (CAT), and glutathione peroxidase (GSH-Px) were quantified using commercial assay kits (Beyotime, China), following manufacturer protocols.

### 2.9 Statistical analysis

All results are reported as the mean ± standard deviation (SD) of triplicate experiments, with error bars representing the SD. Statistical analysis was conducted using SPSS software (version 23, SPSS Inc., Chicago, IL, United States). Differences between groups were assessed using analysis of variance (ANOVA) followed by Duncan’s multiple comparison test, with a significance level set at *p* < 0.05. Asterisk notation: * (*p* < 0.05), ** (*p* < 0.01), *** (*p* < 0.001). Graphs were generated using Origin 9.1 (OriginLab, Northampton, MA).

## 3 Results

### 3.1 Bacteria isolation and identification

**Figure 1:**
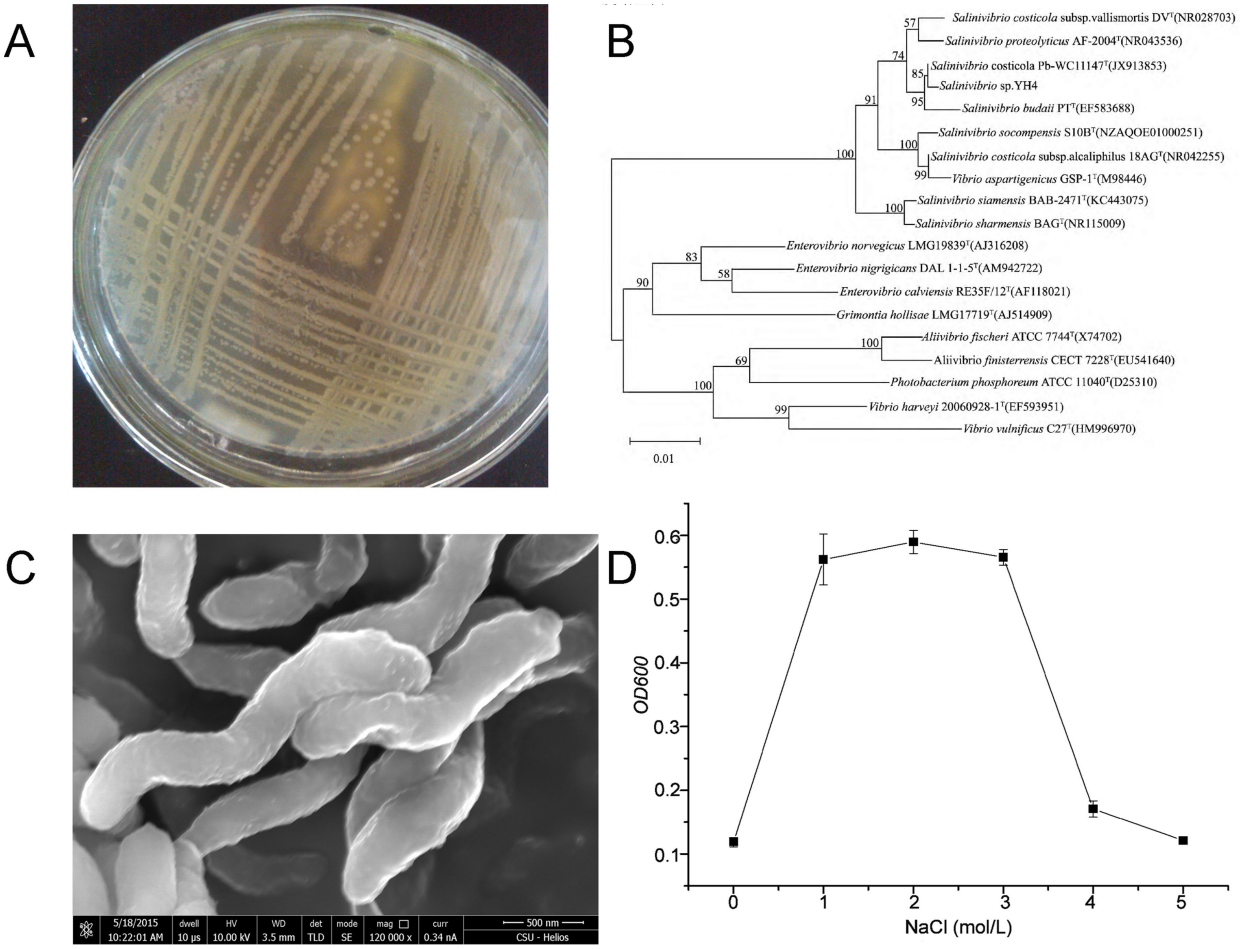
Salt tolerance-linked characterization of *Salinivibrio* sp. YH4. **(A)** Colony morphology on casein agar showing clear proteolysis. **(B)** Phylogenetic tree constructed using maximum-likelihood analysis of 16S rRNA sequences. **(C)** SEM image (×120,000) illustrating vibrioid cell morphology. **(D)** Growth curves under varying NaCl concentrations (0–5 M).

Protease-producing strains were isolated from the hypersaline sediments of Yuncheng Salt Lake using casein-containing screening plates. Strain YH4 exhibited prominent proteolytic activity, as evidenced by clear hydrolysis zones on casein agar (Figure 1A). Scanning electron microscopy (SEM) revealed a vibrioid morphology characteristic of the genus *Salinivibrio* (Figure 1C). The 16S rRNA gene sequence of strain YH4 was analyzed using BLAST against the GenBank database, and homologous sequences from phylogenetically related species were retrieved to construct a maximum-likelihood phylogenetic tree (Figure 1B). The analysis demonstrated that the 16S rRNA sequence of YH4 (GenBank accession: KR870826.1) exhibited the highest similarity (99%) with *Salinivibrio costicola* Pb-WC11147T, confirming its classification within the *Salinivibrio* genus. Growth analysis under varying NaCl concentrations (0–5 M) revealed optimal growth at 1–3 M, with negligible growth outside this range (Figure 1D), thereby confirming *Salinivibrio* sp. YH4 as a moderate halophile.

### 3.2 Extracellular protease profile of YH4

**Figure 2:**
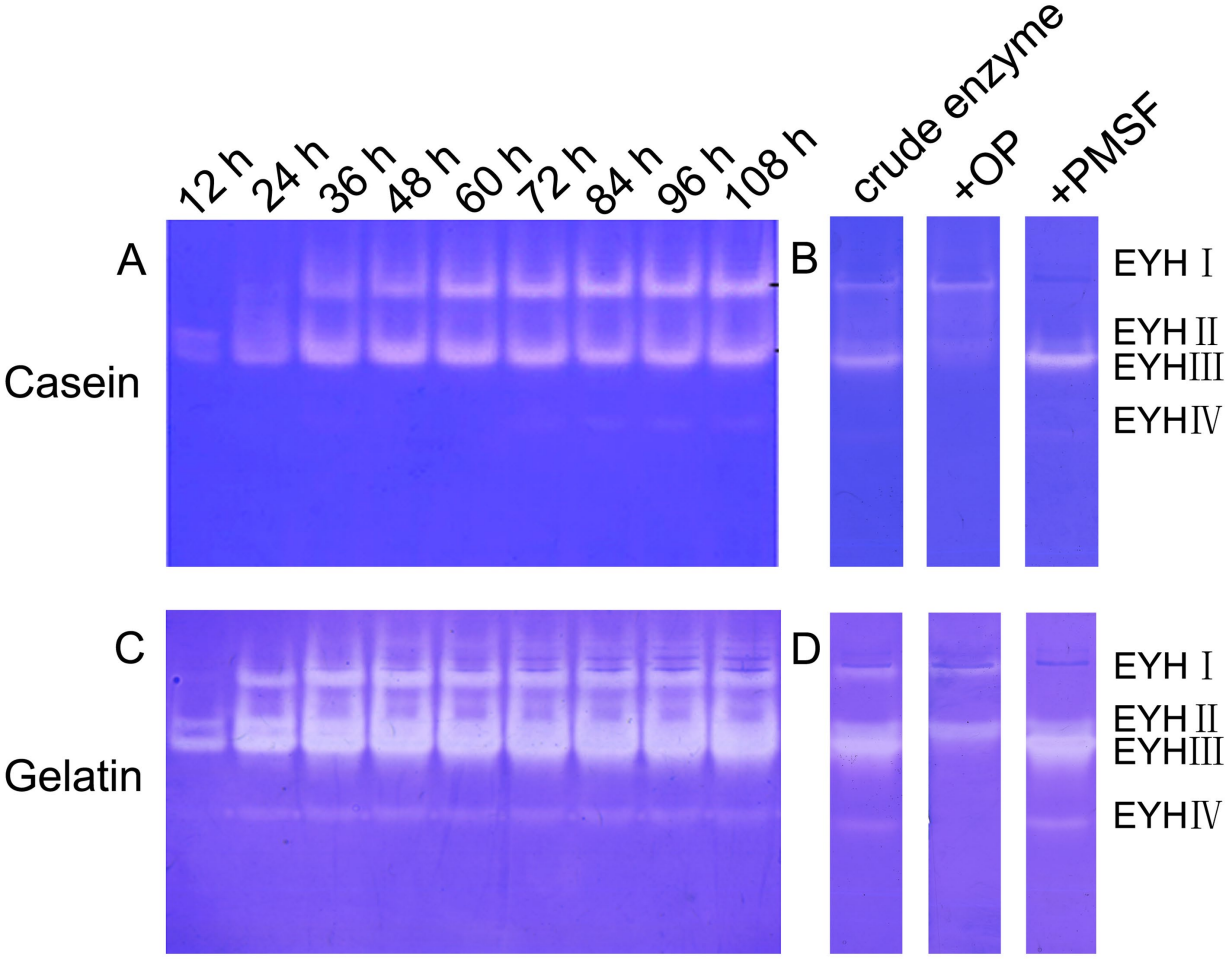
Substrate zymography and inhibitor sensitivity analysis of extracellular proteases from *Salinivibrio* sp. YH4. **(A)** Casein-immersion zymographic detection displaying temporal changes in protease activity during fermentation. **(B)** Inhibitor-treated casein-immersion enzyme profile. **(C)** Gelatin-immersion enzyme detection. **(D)** Inhibitor-treated gelatin-immersion enzyme profile.

Extracellular protease production by *Salinivibrio* sp. YH4 exhibited time-dependent dynamics during fermentation. Protease activity increased significantly after 24 h, reaching a plateau by 84 h (Figure 2A). Zymographic analysis revealed distinct substrate-specific protease profiles. Two dominant proteases, EYHI and EYHIII, were identified through caseinolytic activity (Figures 2A,B), while four proteases (EYHI, EYHII, EYHIII, EYHIV) were identified through gelatinolytic activity (Figures 2C,D). Notably, EYHI and EYHIII exhibited dual substrate specificity for both casein and gelatin, whereas EYHII and EYHIV were specific to gelatin. Inhibition assays further classified these proteases. EYHIII and EYHIV activities were inhibited by 2.5 mM 1,10-phenanthroline (OP), while EYHI and EYHII activities were inhibited by 2.5 mM phenylmethylsulfonyl fluoride (PMSF). These findings demonstrate that *Salinivibrio* sp. YH4 primarily secretes metalloproteases, with EYHIII identified as the predominant metalloprotease, alongside serine proteases.

### 3.3 Purification and identification of protease EYHIII

**Figure 3:**
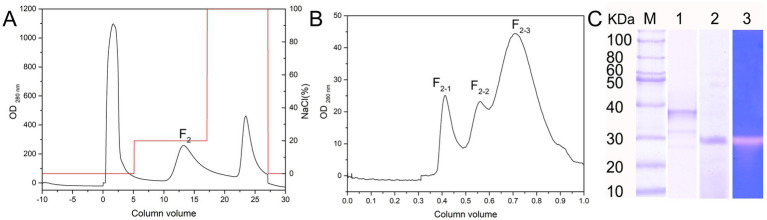
Isolation and purification of protease EYHIII. **(A)** DEAE anion-exchange chromatogram. **(B)** Gel filtration chromatogram of the F2 component. **(C)** Electrophoretic analysis: 1. SDS-PAGE 2. Native electrophoresis 3. Casein-immersion zymographic.

The extracellular crude enzyme extract of *Salinivibrio* sp. YH4 was purified using ammonium sulfate precipitation (50% saturation), dialysis, and sequential chromatography. Initial DEAE anion-exchange chromatography (20 mM Tris–HCl, pH 8.0) with isocratic elution (25% 1 M NaCl) yielded a single active fraction (F2) that exhibited protease activity (Figure 3A). Subsequent purification by gel filtration chromatography (using a Superdex 75 column) resolved three protein peaks, of which only F2-2 displaying proteolytic activity (Figure 3B). SDS-PAGE analysis confirmed that the purified enzyme, designated EYHIII, had a molecular weight of approximately 35 kDa (Figure 3C).

LC–MS/MS analysis of EYHI identified five peptide sequences that were 100% identical to the zinc metalloprotease precursor from *Salinivibrio proteolyticus* (Table 2). Based on these peptide sequences, similar protease sequences were searched in the NCBI database, and the conserved regions were identified. Primers were then designed using the gene sequences from these conserved regions to amplify the corresponding EYHIII protease gene. Three sets of nested primers were then designed, and TAIL-PCR was employed to obtain the 3′ and 5′ untranslated regions of EYHIII. The resulting sequences were assembled and validated. The final full-length enzyme sequence of EYHIII comprised 611 amino acids (GenBank: QAB01367.1). Furthermore, the full-length EYHIII enzyme exhibited 99.51% similarity with the M4 family metallopeptidase from *Salinivibrio proteolyticus* and 79.05% similarity with the M4 family metallopeptidase from *Salinivibrio socompensis*, indicating that EYHIII belonged to the M4 family.

**Table 2:** Amino acid sequence of the protease by mass spectrometry.

Peptide mass	Peptide sequence	Sequence header	Similarity (%)
1995.88	TGQYLYGTDYDDFPVDK	Zinc metalloprotease precursor [*Salinivibrio proteolyticus*]	100
1705.90	AFYLLANKPNWDVR	Zinc metalloprotease precursor [*Salinivibrio proteolyticus*]	100
1737.88	AFYLLANKPNWDVR+Dioxidation (W)	Zinc metalloprotease precursor [*Salinivibrio proteolyticus*]	100
2824.26	YTNGAYSPLNDAHYFGHVVFNMYK	Zinc metalloprotease precursor [*Salinivibrio proteolyticus*]	100
1225.58	YFEQPSRDGK	Zinc metalloprotease precursor [*Salinivibrio proteolyticus*]	100

**Figure 4:**
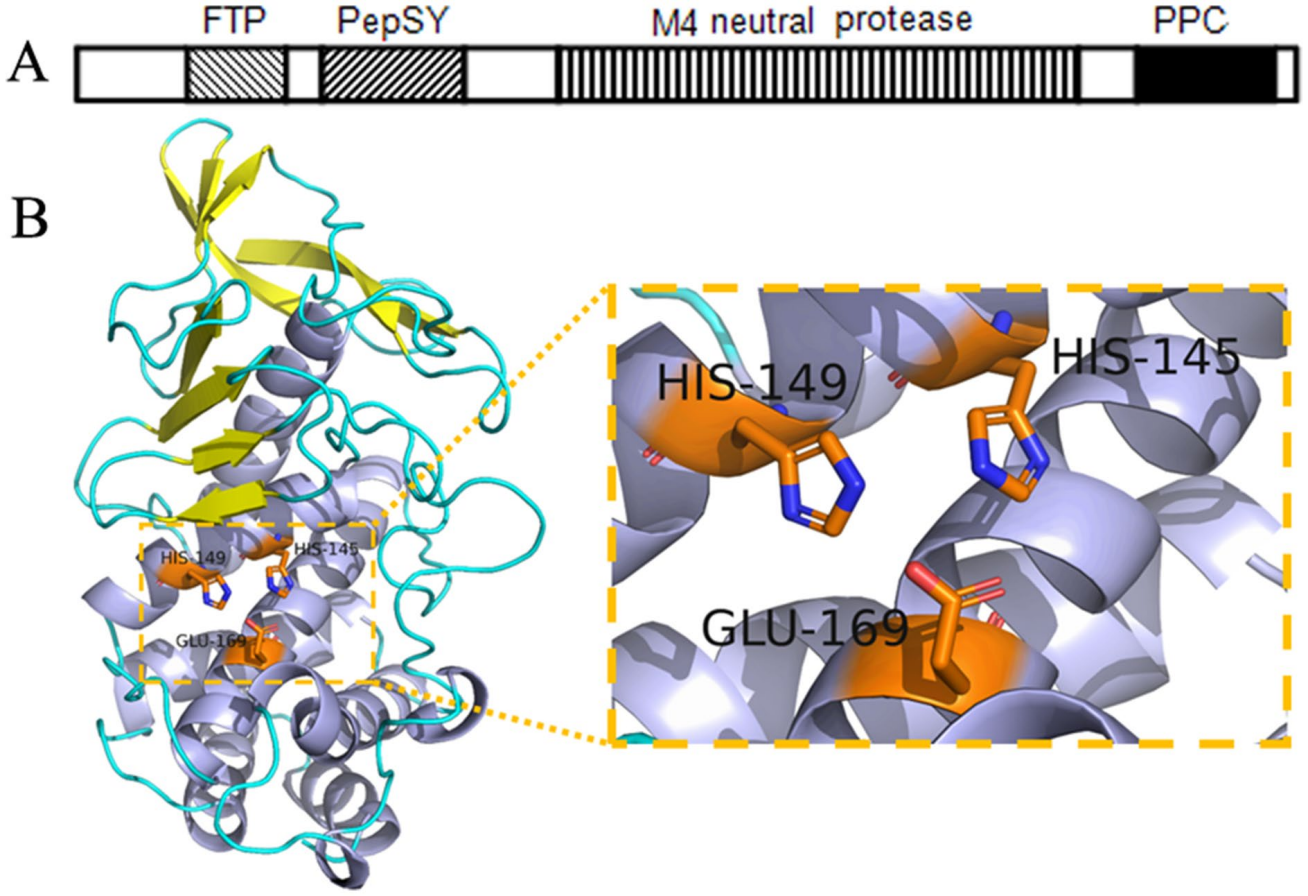
Structural analysis of protease EYHIII. **(A)** Conserved domain architecture of EYHIII. **(B)** Predicted three-dimensional structure of the catalytic domain and the catalytic triad.

BLAST analysis of the conserved domains within the full-length EYHIII enzyme revealed the presence of an FTP domain, a PepSY domain, an M4 neutral protease domain, and a C-terminal PPC domain (Figure 4A). The predicted tertiary structure adopted a heart-shaped configuration, with the N-terminal domain primarily comprised of *β*-sheets and the C-terminal domain dominated by *α*-helices (Figure 4B). Within the catalytic domain, the zinc-binding residues were H345, H349, and E369, forming a proteolytic triad essential for enzyme activity. The Expasy database predicted the molecular weight of protease EYHIII to be 66.67 kDa. However, SDS-PAGE electrophoresis and mass spectrometry analysis of purified EYHIII revealed a molecular weight of 33.3 kDa.

### 3.4 Effects of temperature, pH, NaCl, and metal ions on the activity and stability of protease EYHIII

**Figure 5:**
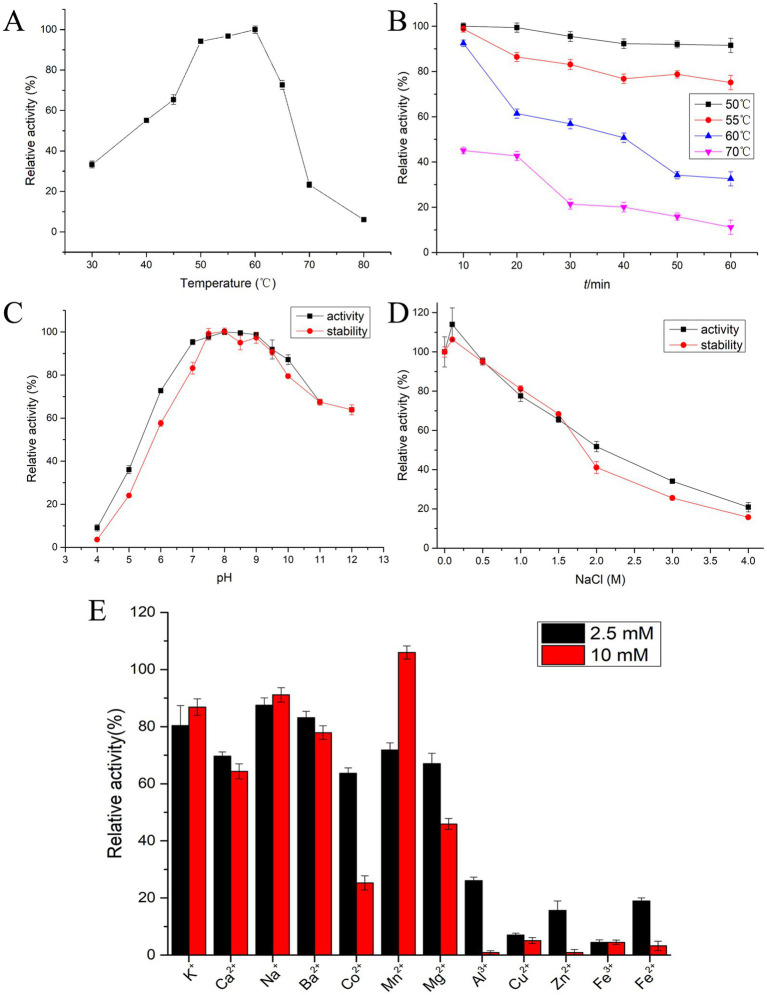
Biochemical characterization of protease EYHIII. **(A)** Optimal activity temperature: EYHIII activity was measured in 20 mM Tris–HCl (pH 8.0) at 30–80°C for 10 min. The highest activity set as 100%. **(B)** Thermal stability analysis: EYHIII was pre-incubated at 50, 60, and 70°C for durations of 10–60 min. And then, the residual activity was measured at standard conditions (50°C, pH 8.0) (100% = activity at 50°C/pH 8.0 without pretreatment). **(C)** Optimal pH and pH stability: EYHIII activity was measured at 50°C across a pH range of 4.0–12.0 using 0.02 M buffers (Citrate-Na₂HPO₄ for pH 4.0–7.0, Tris–HCl for pH 7.0–9.0, Gly-NaOH for pH 9.0–12.0). The highest activity set as 100%. For pH stability, EYHIII was pre-incubated in the respective buffers at 4°C for 24 h, then the residual activity was assayed under standard conditions (50°C, pH 8.0) (100% = activity at 50°C/pH 8.0 without pretreatment). **(D)** Salt effects and halotolerance: Effect of NaCl concentration on the activity of EYHIII was detected at 50°C in different concentrations of NaCl ranging from 0 to 4 M. For salt halotolerance, EYHIII was incubated in NaCl solutions (0–4 M) at 4°C for 24 h. then the residual activity was assayed under standard conditions (50°C, pH 8.0) (100% = activity at 50°C/pH 8.0 without pretreatment). **(E)** Effects of various metal ion concentrations: EYHIII was incubated for 10 min at 50°C, pH 8.0 with 2.5 or 10 mM of various metal ions. Relative to the control (100% activity without metal ions). All assays were conducted in triplicate, with results expressed as mean ± SD.

As shown in Figure 5A, the optimal temperature for protease EYHIII activity ranged from 50 to 60°C, which was consistent with the characteristics of typical mesophilic enzymes. Although its peak activity occurred at 60°C, thermal stability tests revealed a significant gradient in heat tolerance (Figure 5B). After incubation at 50°C for 1 h, the enzyme activity remained largely unaffected, demonstrating both high catalytic efficiency and excellent short-term stability near its optimal temperature. However, stability declined sharply as the temperature exceeded 55°C. For instance, a 20-min treatment at 60°C resulted in approximately 40% activity loss, and prolonged exposure to higher temperatures led to an 80% reduction in activity after 30 min at 70°C.

Figure 5C demonstrated that EYHIII exhibited higher catalytic activity under neutral to slightly alkaline conditions. The enzyme showed optimal activity between pH 7.5 and 9.5 and maintained over 90% of its activity across a pH range of 7.0–10.0. The pH stability results further confirmed that EYHIII primarily exhibited catalytic activity under alkaline conditions, displayed enhanced stability in alkaline environments.

The effects of NaCl concentration (0–4 M) on the enzyme activity of EYHIII illustrated in Figure 5D. The presence of 0.1 M NaCl enhanced protease activity, and at 1 M NaCl, EYHIII retained over 80% of its activity. However, at 4 M NaCl, activity dropped to less than 20%. Notably, after 24 h of incubation in varying salt concentrations, residual activity measurements indicated that EYHIII was highly stable in salt solutions.

Figure 5E depicted the impact of various metal ions on the catalytic activity of EYHIII at different concentrations. While K^+^, Ca^2+^, Na^+^, and Ba^2+^ had minimal effects, the presence of 10 mM Mn^2+^ significantly enhanced the enzyme activity. Conversely, 10 mM Mg^2+^ caused a reduction in activity by over 50%. Co^2+^ inhibited the activity by approximately 35% at 2.5 mM and around 70% at 10 mM, while 2.5 mM Cu^2+^ nearly completely inhibited EYHIII activity. Furthermore, EYHIII demonstrated heightened sensitivity to 2.5 mM Al^3+^, Cu^2+^, Zn^2+^, Fe^2+^, and Fe^3+^.

### 3.5 Substrate specificity of protease EYHIII

We selected casein, gelatin, azocasein, synthetic dipeptides, and both soluble and insoluble collagen to evaluate EYHIII’s substrate specificity. Casein, a major milk protein with a complex structure, mimics natural protease challenges. Gelatin, derived from collagen, is stable, soluble, and demonstrates enzyme performance on modified proteins. Azocasein, a modified form of casein, enables sensitive and quantitative tracking of enzyme activity. Synthetic dipeptides, with their clearly defined structures, help elucidate the enzyme’s catalytic mechanism. Soluble collagen is easier to hydrolyze, while insoluble collagen requires stronger enzymatic activity. This diverse substrate selection highlighted the enzyme’s versatility and potential for various applications.

#### 3.5.1 Catalytic efficiency on synthetic dipeptides

**Figure 6:**
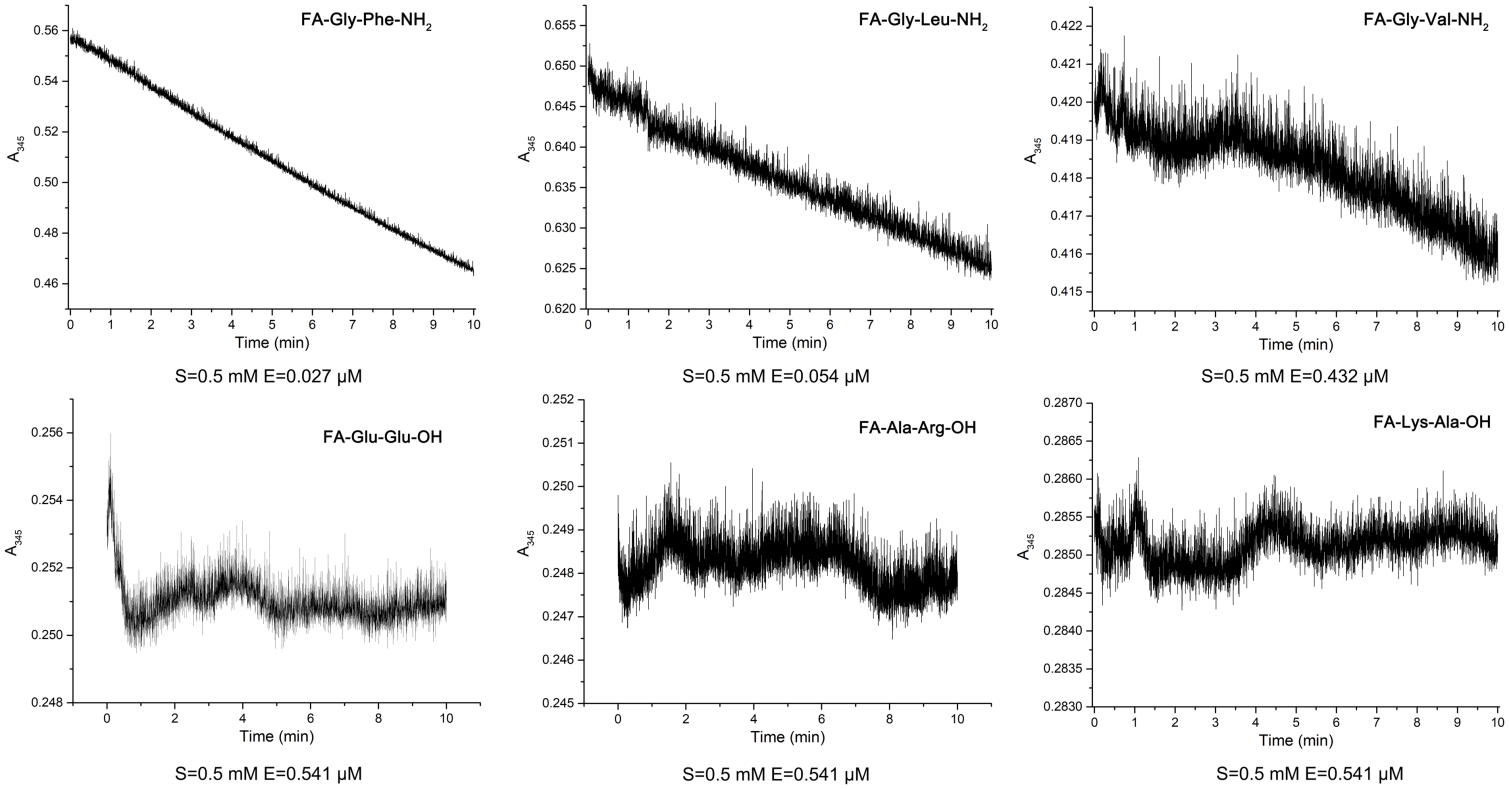
Hydrolysis of six synthetic dipeptides by EYHIII: the dipeptide substrates were initially prepared at 1 mM in 20 mM Tris–HCl buffer (pH 8.0) and then diluted to a final concentration of 0.5 mM in a 200 μL reaction mixture containing 1% DMSO. EYHIII was also diluted in the same buffer, and a 100 μL enzyme solution was mixed with 100 μL of the dipeptide substrate. Following a 30 s equilibration in the cuvette, absorbance at 345 nm (Δ*A*_345_) was recorded at 1 s intervals over 600 s. All experiments were performed in triplicate (n ≥ 3) to ensure statistical reliability.

To elucidate the substrate specificity of protease EYHIII, six synthetic dipeptides bearing furylacryloyl (FA) groups were designed. Proteolytic cleavage of these substrates was monitored in real-time via spectrophotometric detection of absorbance decreases at 345 nm (Δ*A*_345_), enabling calculation of catalytic efficiency (*K_cat_*/*K_m_*) for each substrate (Figure 6; Table 3). EYHIII demonstrated robust hydrolysis of FA-Gly- Phe-NH_2_ and FA-Gly-Leu-NH_2_, which contain large hydrophobic residues (Phe, Leu) at the P1′ position, while showing minimal activity toward FA-Gly-Val-NH_2_, which features the smaller hydrophobic residue Val. Among hydrolyzed substrates, FA-Gly-Phe-NH₂ demonstrated the highest catalytic efficiency (*K_cat_*/*K_m_* = 36.49 mM^−1^ s^−1^), surpassing FA-Gly-Leu-NH₂ by 17.1-fold and FA-Gly-Val-NH₂ by 405.1-fold. In contrast, no hydrolysis was observed for substrates containing acidic (FA-Glu-Glu-OH), basic (FA-Ala-Arg-OH), or neutral (FA-Lys-Ala-OH) residues at P1’ position.

**Table 3:** Comparison of substrate specificity of synthetic dipeptides with EYHIII*.

Dipeptides	FA-Gly-Phe-NH_2_	FA-Gly-Leu-NH_2_	FA-Gly-Val-NH_2_
[E] (μM)	0.027	0.054	0.432
*k*_cat_*/K_m_* (mM^−1^ s^−1^)	36.49	2.14	0.09

*The reaction was carried out in 20 mM Tris–HCl buffer (pH 8.0) at 25°C for 10 min. [E] refers to the final concentration of EYHIII in each reaction mixture. [S] = 0.5 mM.

#### 3.5.2 Activity of EYHIII on protein substrates

Protease EYHIII displayed variable hydrolytic activity toward different substrates, with notable substrate-dependent preferences (Table 4). Among the three soluble protein substrates, casein exhibited the highest susceptibility to enzymatic degradation, with an activity of 39,514 ± 11.1 U/mg. Azocasein was the second most efficiently hydrolyzed substrate, demonstrating significantly lower activity (3,758 ± 0.74 U/mg) compared to casein. Gelatin showed minimal hydrolysis (338 ± 2.50 U/mg), likely due to its partially hydrolyzed and denatured nature, which reduces EYHIII’s ability to cleave peptide bonds efficiently. Among the insoluble collagen substrates tested, bovine collagen BP-SF exhibited the highest relative activity (1907.54 ± 2.51 U/mg), followed by bovine collagen fiber BF-50 (2438.63 ± 1.43 U/mg). Porcine collagen PP-100 demonstrated the lowest activity (1651.26 ± 1.50 U/mg).

**Table 4:** Specificity for different protein substrates.

Soluble substrate	Relative activity^a^ (U/mg)	Insoluble collagen substrate	Relative activity^a^ (U/mg)
Casein	39,514 ± 11.1	Bovine collagen fiber BF-50	2438.63 ± 1.43
Azocasein	3,758 ± 0.74	Pig collagen PP-100	1651.26 ± 1.50
Gelatin	338 ± 2.50	Bovine collagen BP-SF	1907.54 ± 2.51

^a^The specific activity of EYHIII toward each substrate was measured at 50°C and pH 8.0. The data shown in the table are from triplicate experiments (mean ± SD).

#### 3.5.3 Hydrolysis of soluble collagens

**Figure 7:**
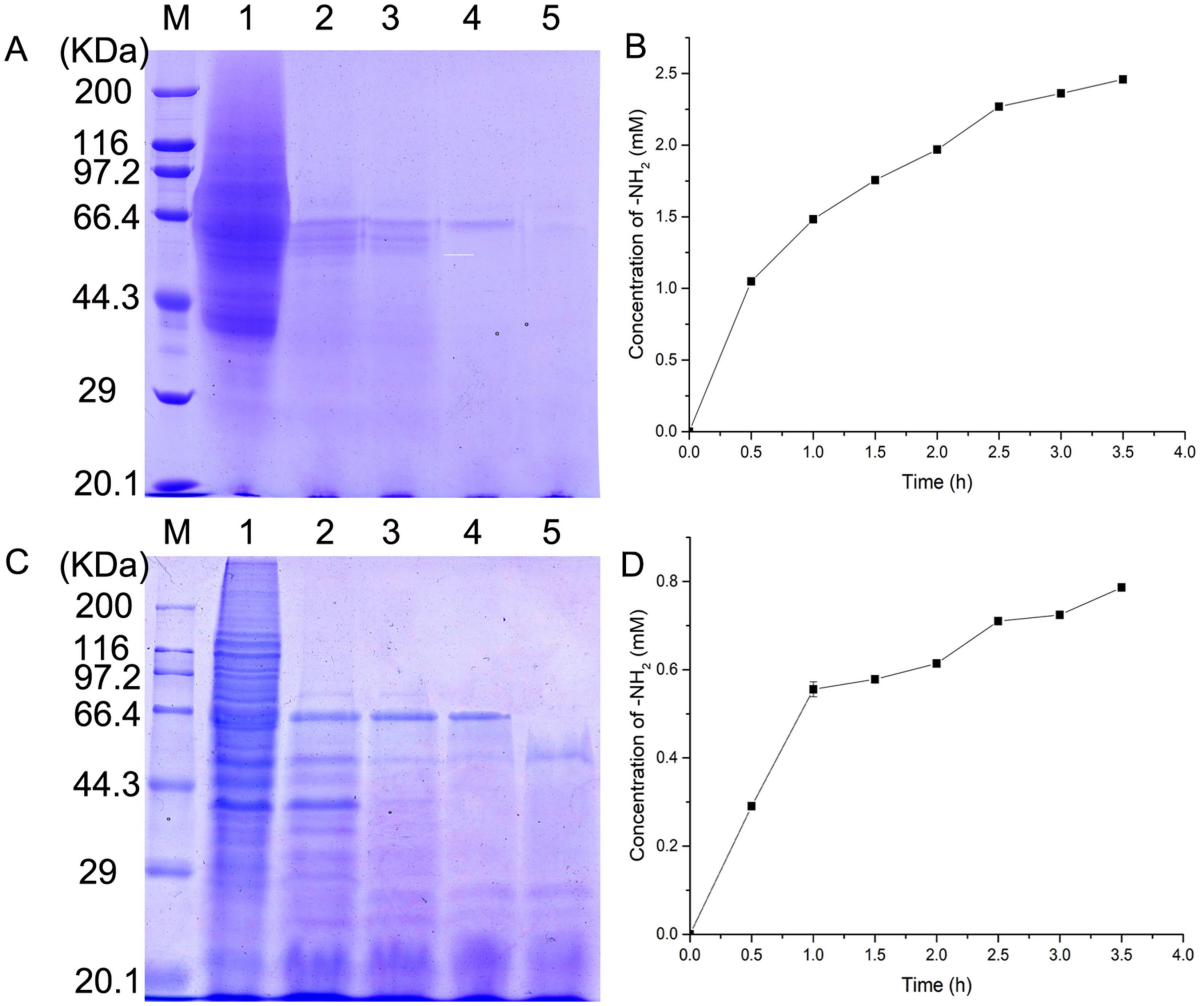
Hydrolysis kinetics of soluble collagens by EYHIII. **(A)** SDS-PAGE Analysis of fish skin collagen hydrolysis: self-extracted soluble fish skin collagen (5 mg/mL) was treated with EYHIII, and samples were collected at10, 20, 30, and 60 min. Lane M represents the molecular weight marker (kDa). Lane 1 shows the untreated fish collagen, while lanes 2–5 display progressively fragmented hydrolysis products. **(B)** Hydrolysis Kinetics of Fish Collagen: Quantitative analysis of fish collagen degradation was performed by measuring the concentration of free amino groups (–NH₂) over time (test per 0.5 h). **(C)** SDS-PAGE Analysis of Porcine Collagen Hydrolysis: Soluble porcine collagen (5 mg/mL) was similarly incubated with EYHIII under identical conditions. Lane M displays the molecular weight marker (kDa), and Lane 1 shows the untreated porcine collagen. Lanes 2–5 represent the hydrolysis products collected at 10, 20, 30, and 60 min. **(D)** Hydrolysis Kinetics of Porcine Collagen: Quantitative analysis of fish collagen degradation was performed by measuring the concentration of free amino groups (–NH₂) over time (test per 0.5 h). All experiments were performed in triplicate (*n* ≥ 3) to ensure statistical reliability.

To further elucidate the substrate specificity of EYHIII, self-extracted soluble collagens from porcine and fish skins were treated with the enzyme at equal concentrations, and hydrolysis kinetics were analyzed via SDS-PAGE (Figure 7). Time-course experiments revealed progressive degradation of both collagens, with fish collagen exhibiting faster fragmentation into low-molecular-weight peptides compared to porcine collagen under equivalent enzyme concentrations (Figures 7A,C). Quantitative analysis confirmed this trend: fish collagen hydrolysis increased rapidly within 0–2.5 h before plateauing, whereas porcine collagen degradation peaked within 1 h and slowed thereafter (Figures 7B,D). After 3.5 h of incubation, the concentration of free amino groups (–NH₂) reached 2.5 mM for fish collagen, compared to 0.8 mM for porcine collagen.

#### 3.5.4 Antioxidant activity of fish collagen hydrolysates

**Figure 8:**
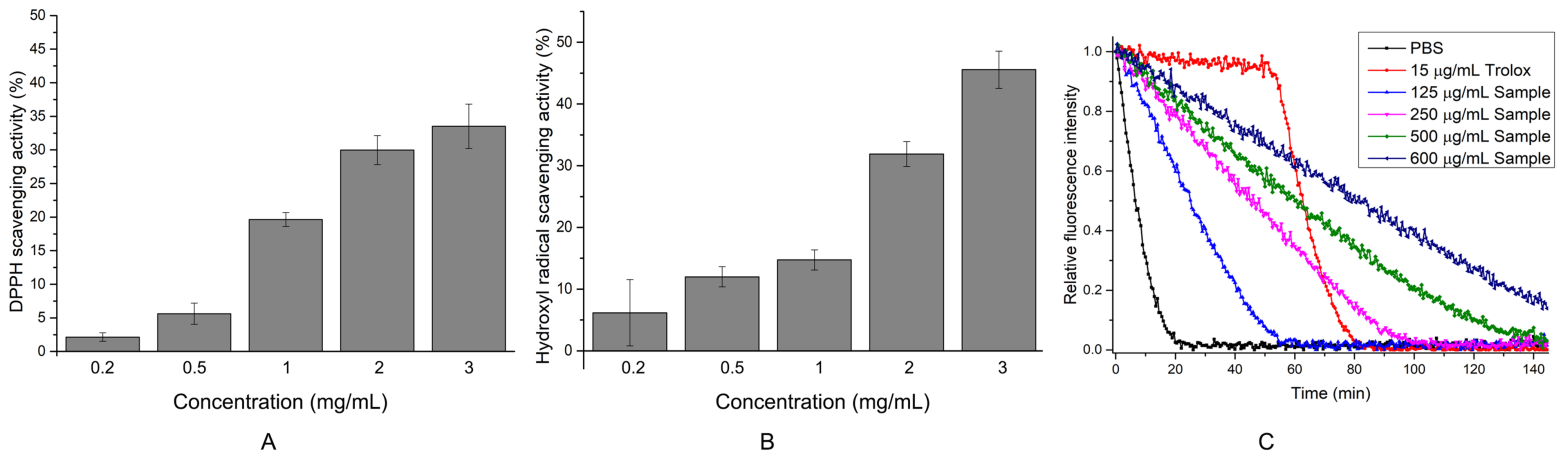
Determination of antioxidant capacity of fish collagen hydrolyzed peptides at different concentrations. **(A)** DPPH radical scavenging activity: Each reaction included 40 μL of peptides (The final concentration is 0.2–3 mg/mL) solution or ddH₂O (control) mixed with 200 μL of 0.1 mM DPPH working solution. After 60 min of incubation at 37°C in the dark, 150 μL of the reaction mixture was transferred to a microplate, and absorbance was recorded at 517 nm. **(B)** Hydroxyl radical scavenging activity was assessed by mixing 40 μL of FeSO₄ (2 mM), 40 μL of 1,10-phenanthroline (2 mM), and 80 μL of peptides (The final concentration is 0.2–3 mg/mL). The reaction was initiated by adding 40 μL of 0.1% H₂O₂ and incubated at 37°C for 60 min in the dark. Following incubation, 150 μL of the reaction mixture was transferred to a microplate for absorbance measurement at 536 nm. Controls included a damage group (without antioxidant) and a blank group (without H₂O₂). **(C)** ORAC: 150 μL of sodium fluorescein solution (96 nM in PBS, pH 7.4) was combined with either 20 μL of collagen hydrolyzed peptides (The final concentration is 125–600 μg/mL), 20 μL of Trolox solution (150 μg/mL, positive control), or 20 μL of 0.01 M PBS (blank). The reaction was initiated by adding 30 μL of preheated AAPH solution (320 mM at 37°C). Fluorescence was monitored every 30 s for 150 min at excitation/emission wavelengths of 485/538 nm. All experiments were performed in triplicate (*n* ≥ 3) to ensure statistical reliability.

Given that EYHIII exhibits lower degradation ability toward porcine skin collagen compared to fish collagen, only the antioxidant activity of fish collagen hydrolysate was evaluated in this study. The fish collagen hydrolysis mixture was lyophilized, and various amounts of the hydrolysate were used to assess its antioxidant activity (Figures 8). At a concentration of 3 mg/mL, the hydrolysate demonstrated DPPH and hydroxyl radical scavenging rates of 33.53 ± 3.30% and 45.55 ± 3.00%, respectively. The peroxyl radical scavenging activity, determined using the ORAC method, was 1.69 ± 0.07 mmol TE/g for the hydrolyzed peptide (Figures 8C). Upon addition of the peptide, the fluorescence decay of sodium fluorescein was significantly delayed. Even at concentrations as high as 0.6 mg/mL, residual sodium fluorescein fluorescence was still observed at the end of the assay. These results suggested that the fish collagen hydrolysate exhibits dose-dependent antioxidant activity, effectively neutralizing multiple radical species (DPPH, OH•, ROO•) and mitigating oxidative damage.

### 3.6 Cytotoxicity and intracellular ROS scavenging by fish collagen hydrolysates in HUVECs

**Figure 9:**
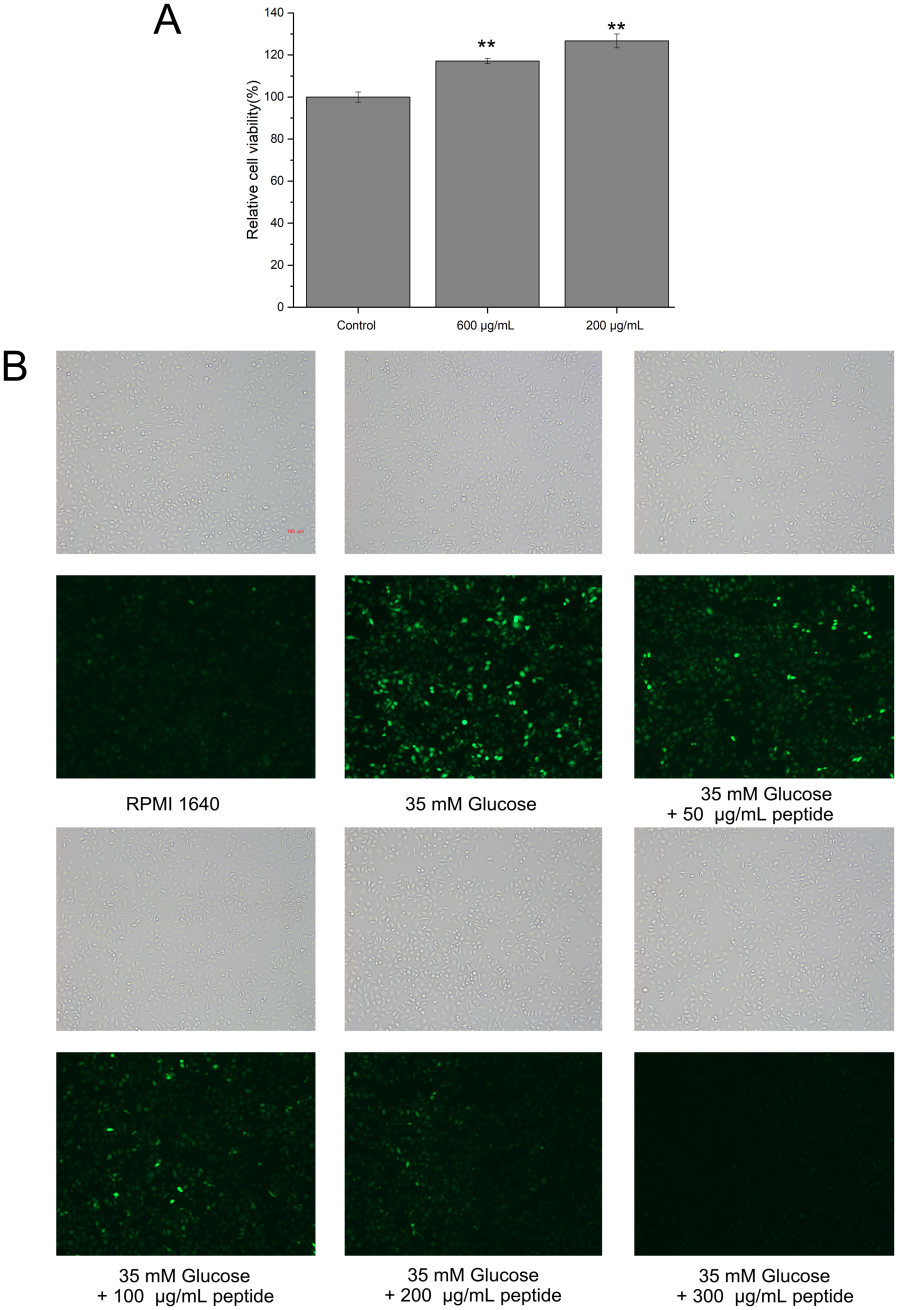
Cytotoxicity and intracellular ROS scavenging activity of fish collagen hydrolysate in HUVECs. **(A)** Relative cell viability tested by 3-(4,5-dimethylthiazol-2-yl)-2,5-diphenyltetrazolium bromide (MTT) assay (*p ≤ 0.05; **p ≤ 0.01; ***p ≤ 0.001). **(B)** The intracellular ROS level in HUVECs in high glucose environment as indicated with green fluorescence by 2,7-dichlorofluorescin diacetate (DCFH-DA) (Scale bar: 100 μm). All experiments were performed in triplicate (*n* ≥ 3) to ensure statistical reliability.

Oxidative damage to endothelial cells is a key factor in the development of various diseases, such as diabetes mellitus and atherosclerosis. Consequently, antioxidant protection of endothelial cells is critical for preventing and treating vascular dysfunction-related conditions. To evaluate the biocompatibility of fish collagen hydrolysates in HUVECs, the MTT assay was employed. In viable cells, intracellular succinate dehydrogenase reduces MTT to form insoluble blue-violet formazan crystals that accumulated within the cells. These crystals were subsequently dissolved in DMSO, and the absorbance was measured at 570 nm. Within a specific range, the absorbance was proportional to the number of viable cells. Treatments with 200 and 600 μg/mL of the hydrolyzed peptides were not only non-toxic but also resulted in a higher cell count compared to the control (Figure 9A), indicating that the peptides were biocompatible within the tested concentration range and suitable for subsequent ROS experiments.

High glucose levels are known to increase oxidative stress in peripheral tissues, leading to the accumulation of intracellular reactive oxygen species (ROS). DCFH-DA, a cell-permeable probe, is hydrolyzed by intracellular lipases to yield DCFH, which is retained within the cell; ROS then oxidize DCFH to generate fluorescent DCF. To evaluate the intracellular ROS scavenging effects of the hydrolyzed peptides, HUVECs were labeled with DCFH-DA. As shown in Figure 9B, cells exposed to 35 mM glucose exhibited a significantly higher DCF fluorescence intensity than the blank control, confirming that high glucose stimulation increases ROS levels and induced an oxidative stress state in HUVECs. This result validated the high-glucose oxidative stress model. Furthermore, treatment with different concentrations of mixed hydrolyzed peptides resulted in a dose-dependent reduction in intracellular fluorescence. Even at 50 μg/mL, the peptides decreased ROS levels; at 100 μg/mL, a greater number of cells exhibited diminished fluorescence; and at 200 μg/mL, the fluorescence intensity was comparable to that of the blank control. At 300 μg/mL, no detectable intracellular fluorescence was observed. These findings indicated that the unpurified mixed hydrolyzed peptides effectively mitigated oxidative damage in HUVECs in a concentration-dependent manner.

### 3.7 Modulation of antioxidant enzyme activities by fish collagen hydrolysates in HUVECs

**Figure 10:**
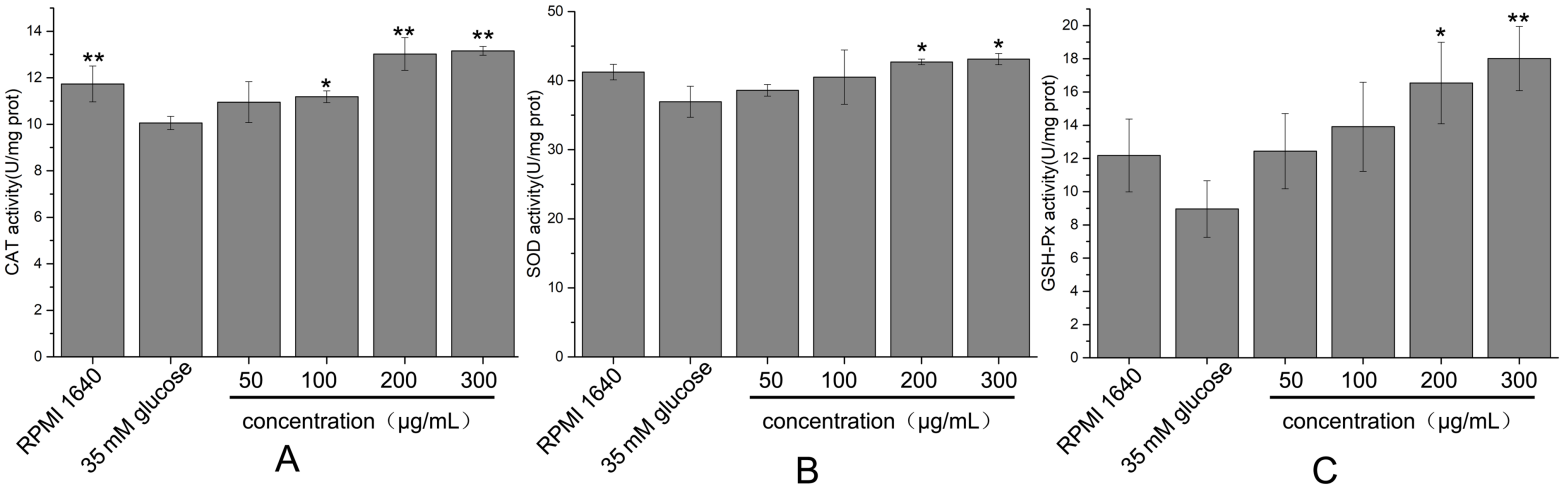
Effects of different concentrations of fish collagen hydrolysate peptides on antioxidant enzyme activity in high-glucose-damaged cells. **(A)** CAT activity. **(B)** SOD activity. **(C)** GSH-Px activity. All experiments were performed in triplicate (*n* ≥ 3) to ensure statistical reliability. Statistical significance was denoted as *p* < 0.05 (*) and *p* < 0.01 (**), based on comparisons with the high glucose-damaged group.

Cells possess a complex antioxidant defense system comprising both antioxidant molecules and enzymes, such as glutathione peroxidase (GSH-Px), superoxide dismutase (SOD), and catalase (CAT). GSH-Px catalyzes the reduction of H₂O₂ and various organic peroxides to H₂O or corresponding alcohols using reduced glutathione (GSH) as a substrate, while SOD converts superoxide anions to H₂O₂, and CAT decomposes H₂O₂ into H₂O. The effects of hydrolyzed peptides on the activities of these antioxidant enzymes in high glucose-damaged HUVECs were shown in Figures 10. In the high glucose-damaged group, the activities of GSH-Px, SOD, and CAT were reduced relative to the blank group. However, only the decrease in CAT activity was statistically significant (*p* < 0.05), whereas the reductions in GSH-Px and SOD activities were not (*p* > 0.05). Treatment with low concentrations of hydrolyzed peptides (50–100 μg/mL) did not result in significant changes in enzyme activities compared with the high glucose-damaged group (*p* > 0.05). In contrast, increasing the peptide concentration to 200 μg/mL led to significant increases (*p* < 0.05) in enzyme activities: CAT activity rose from 85.68 to 110.96%, GSH-Px from 73.57 to 135.79%, and SOD from 87.63 to 103.55%. These findings indicated that high-concentration fish collagen hydrolysate (200 μg/mL) significantly upregulated GSH-Px, SOD, and CAT activities in glucose-stressed HUVECs, counteracting oxidative damage by enhancing endogenous antioxidant defenses.

## 4 Discussion

The isolation of *Salinivibrio* sp. YH4 from Yuncheng Salt Lake underscored the untapped biocatalytic potential of halophilic microorganisms in extreme environments. YH4 was robust growth at 1–3 M NaCl and its secretion of proteases aligned with the adaptive strategies of moderate halophiles, which mitigated osmotic stress through compatible solute synthesis and ion transport (De La Haba et al., 2019). The extracellular protease profile of YH4, dominated by serine proteases and metalloproteases, mirrored trends observed in related *Salinivibrio* strains (Liu et al., 2019b), suggesting evolutionary conservation of hydrolytic functions in saline niches. Notably, *Salinivibrio* sp. YH4 possessed a more complex enzymatic repertoire for gelatin degradation, which likely enhanced its ability to utilize environmental collagen sources. Metalloproteases appeared central to its adaptation to collagen-rich environments.

The full-length of EYHIII enzyme, purified from YH4, exhibited 99.51% similarity with the M4 family metallopeptidase from *Salinivibrio proteolyticus*, confirming its classification within this family. While the ExPASy database predicted a molecular weight of 66.67 kDa for the EYHIII precursor, SDS-PAGE and mass spectrometry revealed a protein size of 33.3 kDa. This discrepancy likely resulted from post-translational processing, a common activation mechanism in proteases. Many proteases were initially synthesized as larger precursors containing signal peptides, prodomains, or inhibitory segments, which are cleaved during maturation or autoproteolytic activation (O'Donohue and Beaumont, 1996; Gao et al., 2010; He et al., 2012).

EYHIII exhibited optimal activity at 50–60°C and pH 7.5–9.5. The enzyme retained over 80% of its activity at 1 M NaCl, demonstrating robust halotolerance. Residual activity assays after 24-h incubation in varying salt concentrations further confirmed its high stability in saline environments, suggesting evolutionary adaptation to the hypersaline conditions of Yuncheng Salt Lake. These properties positioned EYHIII as a promising candidate for industrial applications requiring alkaline and saline resilience, such as leather tanning or seafood waste bioremediation, where conventional proteases underperform (Matkawala et al., 2021; Yan et al., 2022). Although thermal instability above 60°C limits high-temperature applications, its short-term stability under moderate conditions aligns with batch-processing workflows. Notably, EYHIII’s activity was modulated by metal ions: Mn^2+^ activated the enzyme, while Mg^2+^, Co^2+^, and Cu^2+^ inhibited it, suggesting a catalytic mechanism dependent on metal cofactors or ion competition (Ewert et al., 2018). Structural studies are warranted to elucidate binding sites for engineering enhanced robustness.

EYHIII demonstrates robust hydrolytic activity against casein as well as soluble and insoluble collagen substrates. It shared over 99% sequence similarity with vibriolysin, it preferentially hydrolyzed peptide bonds at the P1′ position occupied by hydrophobic residues (Phe, Tyr, Leu) (Miyoshi, 2013). Consistent with this, EYHIII efficiently cleaved substrates featuring large hydrophobic side chains (Leu, Phe) at the P1′ position, displayed diminished activity toward valine-containing substrates, and exhibited no detectable activity against those with acidic (Glu), basic (Arg), or neutral (Ala) residues. This stringent stereochemical preference originates from EYHIII’s structural capacity to accommodate bulky hydrophobic side chains, such as the benzyl group of Phe, while excluding smaller side chains like the isopropyl group of Val and charged residues. This specificity was likely mediated by a hydrophobic S1′ pocket optimized for extended side chains (Wang et al., 2020; Wang et al., 2022). In contrast, porcine collagen exhibited reduced susceptibility to degradation, likely due to species-specific Gly-Pro-X sequence heterogeneity and hydroxyproline modifications that stabilize the triple helix (Shoulders and Raines, 2009; Xu et al., 2019). These findings underscored EYHIII’s broad substrate adaptability and species-selective catalytic efficiency, establishing a mechanistic foundation for its targeted application in marine byproduct biorefining and collagenous waste valorization.

**Figure 11:**
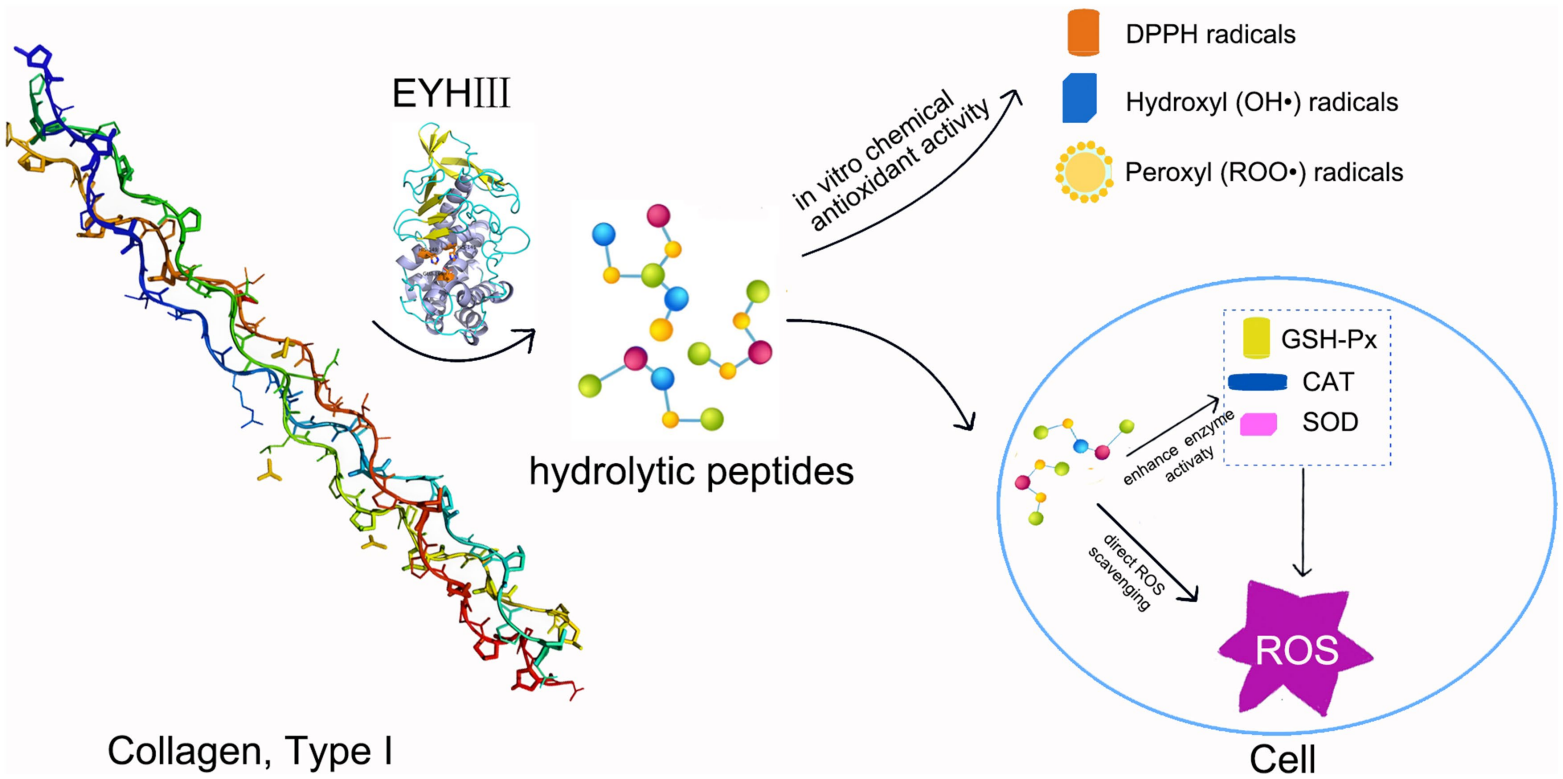
Schematic representation of the antioxidant mechanism of EYHIII. Hydrolysis of collagen by EYHIII, resulting in peptide generation. These peptides could direct ROS scavenging and endogenous enzyme activation to reduced ROS levels in cells. Arrows connecting each stage to emphasize the flow of activity.

This study revealed the significant antioxidant properties of fish collagen hydrolysates generated by EYHIII. At a concentration of 3 mg/mL, the crude hydrolysate displayed exhibited DPPH radical scavenging rates of 33.53 ± 3.30%, hydroxyl radical scavenging rates of 45.55 ± 3.00%, and a peroxyl radical scavenging capacity of 1.69 ± 0.07 mmol TE/g. The antioxidant activity of the hydrolysate was dose-dependent, likely due to the presence of potent hydrogen atom donors capable of neutralizing diverse radical species, including DPPH, hydroxyl (OH•), and peroxyl (ROO•) radicals (Aguilar-Toala and Liceaga, 2021; Zhu et al., 2022). In a high-glucose-induced oxidative stress model using HUVECs, treatment with fish collagen hydrolysates effectively reduced intracellular ROS levels. At 200 μg/mL, the hydrolysates significantly enhanced the activities of antioxidant enzymes (SOD, CAT, and GSH-Px) (*p* < 0.05). This dual mechanism of action, involving direct ROS scavenging and upregulation of antioxidant defenses (Figure 11), was consistent with findings from similar studies. For example, Wang et al. (2016) reported that antioxidant peptides derived from corn gluten meal could neutralize intracellular ROS and enhance antioxidant enzyme activities in Hep G2 cells. Similarly, Chen et al. (2019) observed that a microalgae-derived antioxidant peptide (NDAEYGICGF) mitigated ethanol-induced oxidative stress in HepG2 cells by reducing ROS and increasing SOD and GSH activities. The unpurified hydrolysate in this study indicated robust antioxidant effects. Unlike purified peptides, unpurified mixtures might leverage multiple mechanisms, likely due to synergistic interactions among peptides and auxiliary components, such as free amino acids and other small molecules. Supporting this notion, Masoumifeshani et al. (2025) showed that enzymatic hydrolysis predominantly yielded peptides <3 kDa, which exhibited strong antioxidant activities, including DPPH radical scavenging (78%), ABTS radical scavenging (82%), and FRAP reducing power (74%).

Antioxidant peptides have derived their efficacy from unique amino acid sequences and functional groups (Zhang et al., 2024; Zhu et al., 2024). They neutralized free radicals by donating hydrogen atoms or electrons, and mitigated ROS formation by chelating pro-oxidant metals (e.g., Cu^2+^ and Fe^2+^) or blocking metal-lipid interactions (Bamdad et al., 2015). Additionally, antioxidant peptides activated cellular defense mechanisms through key signaling pathways. Skrzypczak et al. (2017) showed that peptides from *Lactobacillus helveticus* T105 enhanced antioxidant enzyme expression (e.g., SOD, CAT, and GSH-Px) by activating the Nrf2 pathway, thereby increasing cellular antioxidant capacity. Tong et al. (2020) demonstrated that the rice-derived peptide AAGALPS alleviated TNF-*α*-induced oxidative stress and inflammation in vascular endothelial cells by suppressing IKKα activation and stabilizing IκB*α*. Peptides from *Juglans mandshurica Maxim* (TWLPLPR, YVLLPSPK, and KVPPLLY) activated the PI3K/AKT/mTOR pathway, scavenged ROS, restored ATP levels, enhanced GPx activity, and prevented apoptosis in Aβ25-35-treated PC12 cells (Zhao et al., 2020).

The therapeutic potential of fish collagen hydrolysates lies in their ability to reduce oxidative stress and enhance antioxidant defenses, positioning them as promising candidates for managing vascular dysfunction, diabetes, and atherosclerosis (Liang et al., 2019; Ding et al., 2024). The biocompatibility of peptides derived from natural sources like fish collagen further supports their use in antioxidant therapies. Additionally, unpurified hydrolysates were more cost-effective and scalable for industrial applications in functional foods and nutraceuticals. Future research should validate these findings through *in vivo* models and clinical trials. Mechanistic investigations into molecular pathways could deepen understanding of their antioxidant effects, enabling the development of targeted therapies for oxidative stress-related diseases.

## 5 Conclusion

This study highlighted *Salinivibrio* sp. YH4 as a sustainable biocatalytic platform, leveraged its halotolerant metalloprotease EYHIII (>80% activity at 1 M NaCl; pH 7.5–9.5) to convert collagen waste, particularly marine byproducts, into antioxidant hydrolysates. These hydrolysates exhibited dual functionality: direct ROS scavenging and activation of antioxidant enzymes (SOD, CAT, GSH-Px) in oxidative-stressed HUVECs at 200 μg/mL, demonstrated therapeutic potential for metabolic disorders. While EYHIII’s thermal sensitivity and cation inhibition (Mg^2+^/Co^2+^) required structural optimization for industrial use, its collagenolytic efficiency supported circular economy strategies. Future work should prioritize enzyme engineering, mechanistic studies of peptide synergy (via LC–MS/MS), and in vivo validation. Industrially, EYHIII-derived hydrolysates offer promise as natural food preservatives and nutraceuticals targeting oxidative stress-related conditions, bridging environmental sustainability with health innovation.

## Data Availability

The datasets presented in this study can be found in online repositories. The names of the repository/repositories and accession number(s) can be found in the article/supplementary material.

## Footnotes

^1^ https://www.sbg.bio.ic.ac.uk/phyre2/html/help.cgi?id=help/faq
